# An Efficient Robust Eye Localization by Learning the Convolution Distribution Using Eye Template

**DOI:** 10.1155/2015/709072

**Published:** 2015-10-04

**Authors:** Xuan Li, Yong Dou, Xin Niu, Jiaqing Xu, Ruorong Xiao

**Affiliations:** ^1^Science and Technology on Parallel and Distributed Processing Laboratory, School of Computer, National University of Defense Technology, Changsha 410073, China; ^2^Informatization Office, National University of Defense Technology, Changsha 410073, China

## Abstract

Eye localization is a fundamental process in many facial analyses. In practical use, it is often challenged by illumination, head pose, facial expression, occlusion, and other factors. It remains great difficulty to achieve high accuracy with short prediction time and low training cost at the same time. This paper presents a novel eye localization approach which explores only one-layer convolution map by eye template using a BP network. Results showed that the proposed method is robust to handle many difficult situations. In experiments, accuracy of 98% and 96%, respectively, on the BioID and LFPW test sets could be achieved in 10 fps prediction rate with only 15-minute training cost. In comparison with other robust models, the proposed method could obtain similar best results with greatly reduced training time and high prediction speed.

## 1. Introduction

Eye localization is essential to many face analyses. In analysis of the human sentiment, eye focus, and head pose, the location of the eye is indispensable to extract the corresponding information there [1]. In face tracing, eye localization is often required in real time. In face recognition, many algorithms ask for the alignment of the face images based on eye location [2]. Inaccurate location may result in the failure of the recognition [3, 4].

**Figure 1:**
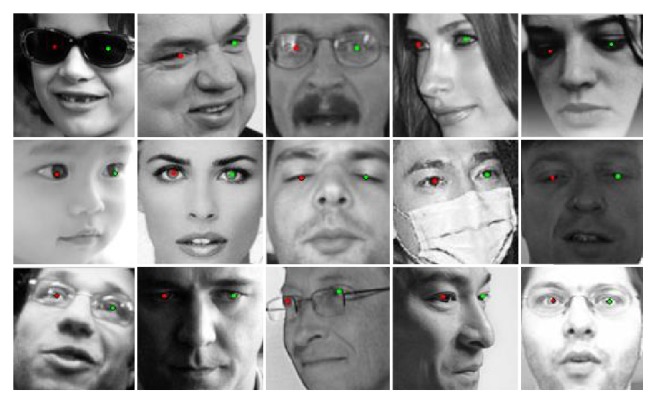
Eye localization result using our method.

However, real-world eye localization is filled with challenges. Face pictures are commonly taken by a projection from the 3D space to the 2D plane. Appearance of the face image could be influenced by the head pose, facial expression, and illumination. Texture around eyes is therefore full of change. Moreover, eyes may be occluded by stuffs like glasses and hair, as shown in Figure 1. To work in any unexpected cases, the algorithm should be robust to those impacts.

In the design of the eye localization algorithm in practical use, prediction accuracy, rate, and the training cost are the most concerned factors. A robust algorithm should keep high prediction accuracy for varying cases with diverse face poses, facial expressions in complex environment with occlusion, and illumination changes. For real time applications, high prediction rate is required. For some online learning systems like the one used for public security, short training time is also in demand to quickly adapt the algorithm to different working places. Low training cost is also of benefit for the tuning of the algorithm. To improve the accuracy in the difficult environment, complex model is often applied. However, the over complicated model will increase the training cost and the prediction time. How to select an approach with enough complexity to achieve high prediction accuracy, high prediction rate, and low training cost at the same time is still a challenge.

Eye localization approaches could be mainly divided into the texture based and the structure based. Texture based methods [5–8] learn the features from the image textures. For the methods exploring local textures [5, 6], high prediction rate could be achieved with simple training. However, they are usually not robust to the situation with occlusion and distortion due to the limited information from the local area. On the other hand, methods like [7, 8] study the global texture feature from entire face image by convolution networks. High prediction accuracy could be obtained by these approaches with high prediction rate. However, the training cost becomes considerable. A long training time is required due to a large number of the model parameters. Proper selection of the model parameters often needs repeated test as well. The structure based approaches [9–12] explore the predefined critical facial points. Eye locations could be detected mainly by the structure information. Although high accuracy could be achieved by a simple training, the prediction often involves an iterative optimization. And the iteration times and predication accuracy highly depend on the initialization. Therefore, the prediction accuracy and rate are usually not stable.

In this study, it was found that there is regular response distribution on the convolution map generated by eye template. This distribution reflects the spatial relationship among the major facial objects. By a nonlinear learning model, such distribution could be explored to predict the location of the eyes. Instead of using local response like conventional template methods, global information could be explored according to such distribution. In this way, eye locations could be accurately predicted and even occlusion or distortion occurs. Besides, high prediction rate could be expected with a noniterative prediction model. To this end, the convolutional networks [7, 8] could be explored. However, conventional convolutional networks learn from the raw image, which may need more layers to map the complex nonlinear relation between the face image and eye locations. On the other hand, convolution map produced by eye template contains concise distribution information, where different facial objects have stable response patterns. Since many unnecessary textures are ignored, distribution could be learned using relatively shallow networks and the training time can be much reduced.

Based on this principle, an efficient and robust eye location algorithm is proposed in this paper. The algorithm explores the face convolution map by the eye template using a BP network. To enhance the performance of the eye template, a novel template training method was proposed by noise suppression control. Besides, a FFT- (fast Fourier transform-) based convolution approach was designed to further improve the training and prediction speed. Eventually, the proposed algorithm could achieve accuracy of 98% and 96%, respectively, on the BioID [13] and LFPW [14] test sets with a prediction rate of 10 fps. However, the training time was only 15 minutes for 13,466 samples.

The rest of the paper is organized as follows.  Section 2 reviews the related work on eye detection.  Section 3 describes the finding of the convolution distribution.  Section 4 gives a detailed description of the proposed approach.  Section 5 discusses the experiments and results. Conclusion is given in Section 6.

## 2. Related Work

Eye localization techniques have been greatly developed in recent years. According to the properties of the explored features, methods could be mainly divided into two classes: the structure based and the texture based approaches.

The structure based approaches explore some critical points on a specific face structure model for the prediction. Typical structure based approaches include ASM [9] and AAM [11]. In these methods, eye locations are estimated by the structure information and the local texture around the points through an iterative process. The iteration time and the prediction accuracy are affected by the initial structure. Moreover, the optimization employs a least square method whose robustness is poor to the case with occlusion.

The texture based approaches extract the face texture to predict the locations of the eyes. Typical structure based approaches include [5, 7, 8, 15]. Instead of using limited critical points by the structure based approach, all the textures from the entire face image could be explored. Previous studies [7] have showed the robustness of the face textures to occlusion and distortion. The texture based approaches are usually noniterative. For the extraction of the texture features, approaches like convolution [5, 7], LBP [16], HOG [17], HAAR [18], and so forth are commonly used. Within them, best prediction accuracy has been reported by the the convolution based approach [7, 8]. Like other texture extraction, convolution operation is also computation intensive since the whole image needs to be scanned over by a texture window. However, it was also found that the convolution feature extraction can be well accelerated in frequency domain by FFT.

Currently, there are mainly two kinds of eye localization using convolution feature extraction. Methods like [5, 19, 20] tried to explore the peak response by a predefined template (as the convolution kernel) with an ideal eye pattern. Since the convolution response of the eye template on the other facial objects like cheek or lip was hard to explain, the remaining part of the response map was ignored, and eyes were only located by the peak response. These approaches have low training cost and high prediction rate. But they are not robust to the occlusion and changing face pose, since the response on the eye by such fixed eye template will greatly decrease in these situations. Prediction by the peak response may eventually fail in these cases. In contrast to exploring the peak response by the complex eye template, other methods try to code the face by the templates of basic image elements, most of which are line segments. Relation between the eye locations and the code distribution of the face could be learned by nonlinear models. Due to the use of global information, the prediction accuracy could be improved and the effects of occlusion and distortion by pose changes could be relieved. However, such nonlinear relationship is usually complex. Models used in those approaches such as Deep CNN [7, 8] are commonly with a large number of parameters, which leads to a costly training phase and a low prediction rate. Moreover, search of the optimal setting of these complex models is also difficult.

In this paper, it was found that there is relative stable global distribution pattern in the convolution response generated by the eye template and the face image. Based on this phenomenon, a novel eye location approach is proposed. This approach explored the global face information produced by the eye template for location prediction to avoid arbitrary decision by only the peak response. Meanwhile, using specific eye template rather than the randomly learned kernels, the convolutional network could be greatly simplified with only one layer. Properties of the response distribution as the new finding will be described in Section 4.  Section 5 gives the proposed algorithm based on these findings.

## 3. Regulation on the Convolution Response Map by Eye Template

The global information of the convolution response map by eye template has long been ignored, since the response distribution of the face is not explicit. Therefore, only the peak response was evaluated to indicate the location of the eyes, as the eye templates normally have the largest convolution response value at the place of the eyes. However, these approaches are usually with low prediction accuracy in practical use. The facial expression, occlusion, and changing pose will all affect the appearance of the eyes, which will lead to lower response there. Moreover, it is also difficult to distinguish the left eyes from the right.

However, it was found in the experiments that the convolution response map of the face image by eye template has regular global distribution, where the facial objects like nose and mouth have relatively stable patterns.

To demonstrate this phenomenon, an experiment which tests the similarity of the response samples from the same facial object has been conducted. KL distance [21] was used to quantitatively measure the similarities of those response patterns as follows:

(1) $$\begin{array}{c} \text{KL}\left(p||q\right)=\underset{\nu \in \Omega }{\sum }p\left(\nu \right)\ln\frac{p\left(\nu \right)}{q\left(\nu \right)}, \end{array}$$

where *p* and *q* are the two test distributions. KL tends to be small when *p* and *q* are similar.

**Figure 2:**
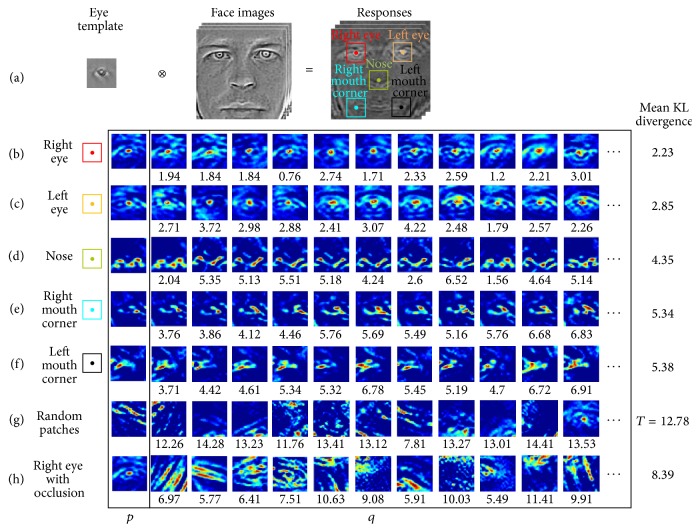
Convolution responses for different facial objects.

In the experiments, 100 front face images were randomly selected from the BioID test set. They were scaled to 128 × 128 and preprocessed to remove the shadow effects by Tan-Triggs [22]. 100 convolution response maps were further generated by a pretrained eye template as shown in Figure 2(a). Five comparison groups (rows (b), (c), (d), (e), and (f) in Figure 2) were defined by the cropped texture samples at five locations (with the color squares and center points) of the left eye (red), right eye (orange), nose (green), left mouth corner (blue), and right mouth corner (black). A texture sample was picked up from each group as the reference to compare with the other samples in the same group with KL distance. In KL measurement, *p* (in the first column of Figure 2) was used for the reference texture and *q* for the texture samples in the same group. *p* and *q* were all normalized to [0,1]. Zero value is replaced by 10^−9^ to avoid the zero division. To demonstrate the similarity of the samples in each facial object group, a group (row (g) in Figure 2) with randomly cropped textures over the face images was also prepared for comparison. Additional group (row (h) in Figure 2) formed by the samples from the right eye with occlusions was prepared for study as well. Colors were used to enhance the visibility of the response maps with the increasing value from blue to red.

From Figure 2, stable responses of the eyes, noses, and mouths ((b)–(g)) can be observed. The average KL distances of these groups were about 2.23, 2.85, 4.35, 5.34, and 5.38, which were significantly smaller than those of the random group *T* = 12.78. This indicates the similarity of the response patterns of the samples from the same facial object.

From Figures 2(b) and 2(c), it can be observed that the distributions of the textures of the left and right eyes are stable unimodal. However similarity between the two eyes is also evident, which implies the difficulty to tell the difference from the left to the right using only the peak response. The distribution of nose responses in Figure 2(d) is multimodal with the peak values in the half part of the stable *M* pattern. The average KL distance in nose group is relatively bigger than that of eyes, which indicates more diversity in this area. The distributions of the left (Figure 2(e)) and right (Figure 2(f)) mouth corners also showed regular unimodal patterns with more variation than that of the mouth. As illustrated in Figure 2(h), the distribution of the samples from occluded eye has irregular pattern and is more similar to the randomly selected patches in group (g) with the average KL distance 8.39.

From the above experiments, it could be noted that a relative stable global distribution pattern could be achieved using convolution by the eye template. The convolution responses of the facial objects have distinctive and stable patterns. These properties could be explored to form a stable spatial relationship between the eye locations and the positions of other facial objects. Even when the information of part of the face is destroyed by the occlusion or distortion, the location of the eyes could also be predicted from the rest of the stable patterns.

## 4. Architecture

**Figure 3:**
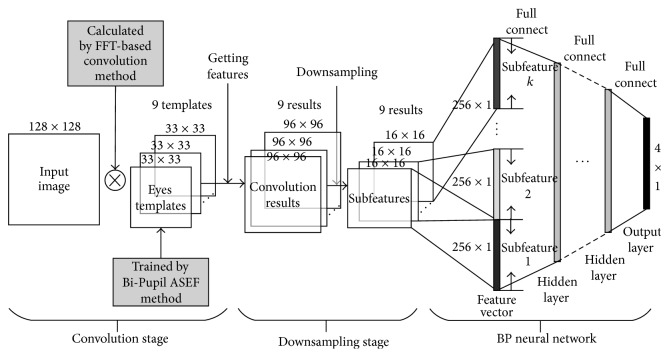
Architecture of the proposed eye localization model.

The architecture of the proposed eye location model mainly consists of 3 stages, that is, convolutional feature extraction, downsampling, and a BP neuronetwork as illustrated in Figure 3. In contrast to many other convolutional networks, only one convolution layer was employed in our work. Moreover, the pretrained eye templates were used as the convolution kernels. Reduced convolution layers could simplify the model and significantly short the training time, while the feature extraction ability may be limited. However, it was found that by proper preprocessing and selection of the convolution kernels, the relationship between the eye locations, and the convolution response could be well learned by a BP network. For image preprocessing, the Tan-Triggs method was selected to reduce the illumination effects. The eye templates were trained by a proposed Bi-Pupil ASEF approach. Convolution operations were implemented by a FFT-based method to further reduce the computation. Details of these approaches are given in the following subsections.

### 4.1. Eye Template Training by Bi-Pupil ASEF

The eye templates are trained by a proposed Bi-Pupil ASEF which is based on ASEF [5]. In ASEF, an eye template sample could be given by each eye image sample with a specific response function. The eye template could be obtained by averaging the template samples to stress the features in common. At the location of the eyes, peak response could be normally observed by the ASEF templates. To efficiently synthesize the characters of the left and right eyes for feature extraction, a modified response function was explored in the proposed Bi-Pupil ASEF. And a multieye template set was employed to cope with the change of scale and rotation.

#### 4.1.1. Bi-Pupil Response Function

It was found the response maps generated by the left and right eyes have certain relations. To efficiently generate response maps and reduce the cost of the separate training of the two eyes, a Bi-Pupil response function has been proposed instead of the one used in ASEF. Consider

(2) $$\begin{array}{c} g\left(x,y\right)=e^{-\left({\left(x-x_{le}\right)}^{2}+{\left(y-y_{le}\right)}^{2}\right)/\sigma^{2}}+e^{-\left({\left(x-x_{re}\right)}^{2}+{\left(y-y_{re}\right)}^{2}\right)/\sigma^{2}}, \end{array}$$

where (*x*_le_, *y*_le_), (*x*_re_, *y*_re_), respectively, give the positions of the left and right eyes. By this function, similar high responses could be achieved on both eyes. The convolution response map could be taken as the average of the ones by left and right eyes templates. However, the training task is reduced to only one template for two eyes. Moreover, by average of the two eyes, the coupling of the template to certain samples could be further decreased. As the conventional ASEF, the Bi-Pupil ASEF eye template training is noniterative. With the increased number of the training samples, the template eventually converges. Therefore, the well trained eye template could also be used to other data sets.

#### 4.1.2. Change Invariant Multieye Template Set

**Figure 4:**
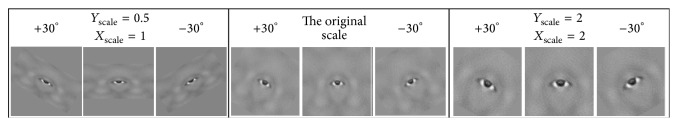
Multieye template set.

To make the template invariant to the changes like eye closing, scaling, and rotation in the real-world use a multieye template set was generated with 3 rotation types in 3 scale levels as illustrated in Figure 4. Accordingly, nine convolution response maps were produced to code the face with the concerns of these situations.

### 4.2. FFT-Based Convolution Method

**Figure 5:**
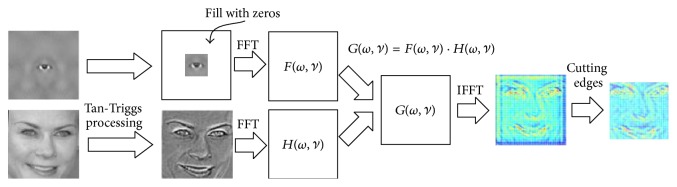
The process of the FFT-based convolution.

In the ASEF based feature extraction, the convolution of the face image and the eye template is implemented by dot product in the frequency domain through FFT. Although FFT could reduce the computation for convolution, it will also bring the frequency effects on the image edge, where the response may be wrongly computed with the periodic edge information from the other side. To mitigate such effects, a cosine window approach [5] has been proposed. However, it was found such approach may fail due to the greatly reduced eye texture when the eyes are not in the central zone of the image. To reduce the computation for convolution operation without introducing additional errors in practical use, a modified FFT-based approach was proposed as illustrated in Figure 5.

We use a zero filled window function to reduce the frequency effects. Comparing to the cosine window method, the zero filled window function is applied on the eyes templates other than the face images. This method can avoid the reduction of the eye texture which happens in the cosine window method. To keep the feature extraction ability and to reduce the frequency effects, the size of zero filled area on the eye templates should be chosen carefully. Based on our experience, this method, that is, only keeping the eyes texture and filling the other area with zeros on the templates, is a good choice. In our experiment, the size of the face images is 128 × 128, and the size of the eye region is approximately 33 × 33. According to the size of the eye region, only the 33 × 33 center area of eye templates is retained.

After the fringe of the eye template is zeroed out, the zero filled eye template and the preprocessed eye image are transformed by FFT. With an IFFT on the dot product of the two frequency maps, an initial convolution map could be achieved. By cutting out the inaccurate edges with a width of 16 pixels, the final convolution result of 96 × 96 is obtained. Such result is the same to that by normal convolution.

To demonstrate the effect of the FFT-based approach for reducing the convolution computation, a computation complexity analysis is given as follows. The analysis is only based on the multiplication, since it is the most time-consuming operation. Given *N* the face image width and *M* the eye templates, the total number of multiplications in a normal convolution in the spatial domain is

(3) $$\begin{array}{c} C_{spatial}\approx O\left(M^{2}{\left(N-M+1\right)}^{2}\right). \end{array}$$

With 3 FFT (two FFT and one IFFT) and the product of the complex matrices in frequency domain, the multiplication number of the FFT-based convolution could be

(4) $$\begin{array}{c} C_{freq}\approx O\left(3N^{2}\log N+4N^{2}\right). \end{array}$$

As in this experiment, *N* = 128 and *M* = 33, for one convolution map, about 10,000,000 multiplications are required for the normal convolution and 40,0000 for the FFT-based approach. Considering 9 maps by the multieye templates, the reduction of the computation is significant. Decreasing of the training and prediction time could benefit from such reduction of convolution computation.

### 4.3. The BP Network and the Cascade Enhancement

Before the further processing, the convolution response maps were firstly normalized with the mean 0 and the standard deviation 1.0. It was found such normalization is significant to improve the prediction accuracy, since the unbalance between the distributions of the response maps could be well reduced. To improve the invariance of the extracted features, max pooling was employed to downsample the normalized response maps to 16 × 16. The nine downsampled matrices were further vectorized and catenated as a vector with the length of 2304 to input to the BP network. The fully connected BP network employed the sigmoid activation function as illustrated in Figure 3. As the output, the location coordinates were also normalized into [0,1]. The BP network is trained by conjugate gradient descent with the least square error between the label and prediction as the error function. By comparison, a 4-layer network with the hidden neurons of [30-20-20-10] was selected considering the prediction accuracy and training time.

**Figure 6:**
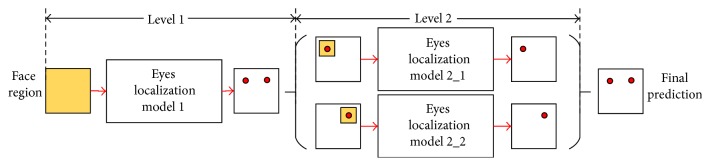
The structure of the two-level cascade enhancement.

To further improve the prediction accuracy, a two-level cascade enhancement scheme has been proposed as illustrated in Figure 6, just as [7] does. The first level gives the initial positions of the eyes from the entire face image. Then, the location of each eye was revised in the second level within a square centered by the initial position. The width of the square is one-fourth of the face region.

The second level only learns the deviation Δ*x* between the prediction by the first level and the labeled location. Since each model in the second level only learns one eye, the single eye response function in original ASEF was used to generate the eye template in the second level. The final prediction *x* is the sum of the output of the first level *x*_1_ and the second level Δ*x*.

## 5. Experiment

The data used for this experiment is the same as [7]. The data set consists of 13,466 training and 2,551 test images. The training samples were selected from the LFW [23] data set and internet. The test set was formed by the complete BioID and LFPW data sets. The BioID data was mainly composed of the regular front images, while the other data such as LFPW were collected from many complex environments including wide changes in head pose, illumination, scale, clarity, and occlusion. Therefore, a robust algorithm should have high accuracy on the LFPW data set. All the images were marked with face region, the locations of the eyes, and nose and two mouth corners.

For evaluation, a relative prediction error is defined as follows:

(5) $$\begin{array}{c} \mathrm{err}=\frac{\sqrt{{\left(x-x_{t}\right)}^{2}+{\left(y-y_{t}\right)}^{2}}}{l}, \end{array}$$

where (*x*, *y*) and (*x*_*t*_, *y*_*t*_) are, respectively, the predicted position and the ground truth and *l* is the biocular distance. Based on this error, the accuracy of the algorithm could be defined as the ratio of the samples, which have the prediction error less than 0.1, to the whole test samples. The mean error is also used as another indicator to evaluate the prediction results in the following experiments.

The proposed algorithm was implemented in Matlab 2014a, and the experiments were conducted on a desktop computer with a 3.3 GHz CPU.

### 5.1. Effects of the Improvement Schemes

In this section, the effects of the above-mentioned improvement schemes will be discussed.

#### 5.1.1. Effects on Prediction Accuracy

In the following experiments, the schemes of the Tan-Triggs preprocessing, Bi-Pupil ASEF eye template training, normalization of the convolution response map, and the cascade enhancement schemes on the prediction accuracy were tested. The proposed model with all the schemes was noted as the standard configuration. As show in Table 1, four compared configurations were prepared: Tan-Triggs is replaced by log(*x* + 1) function (M1), where *x* is the pixel values. Bi-Pupil ASEF is replaced with the original ASEF (M2) which train templates using only right eye location, response map normalization is replaced by no action (M3), and the cascade enhancement is replaced by the only first-level raw model (M4).

As demonstrated in Table 1, all the schemes could effectively improve the prediction accuracy. Using Tan-Triggs rather than the Log function to reduce the illumination effects could increase the accuracy by about 1.5%–2%. The Bi-Pupil ASEF templates can raise the accuracy by about 2%–3% than the original ASEF templates. Response normalization was also of benefit to the accuracy increment about 0.5%–1.5%. In comparison with the raw model, the cascade enhancement could bring 3%-4% accuracy improvement.

#### 5.1.2. Acceleration by FFT-Based Convolution

**Figure 7:**
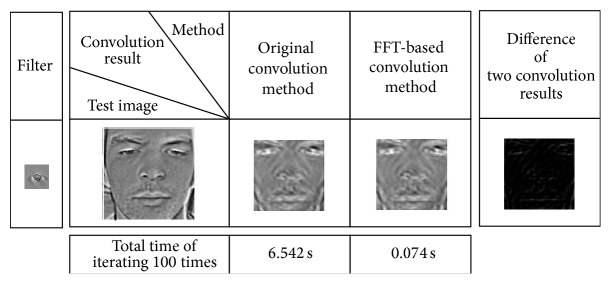
Comparison between the original convolution and the FFT-Based approach.

To demonstrate the performance of the FFT-based approach, a convolution of a face sample and an eye template has been repeated 100 times. As illustrated in Figure 7, there is slight difference between the results by the FFT-based convolution and the direct convolution. However, such difference hardly has any effect on the further analysis with the extracted feature. For the computation efficiency, with the reduced operations, the FFT-based approach could generate almost 80 speed-up ratio in this experiment. For the models with a large numbers of convolution, this approach could significantly decrease the training and prediction time.

### 5.2. Comparison with Other Approaches

To demonstrate the efficiency of the proposed method, comparisons with many state-of-the-art eye location approaches including the texture based and the structure based were conducted considering the prediction accuracy, prediction rate, and training time.

For the texture based approaches, ASEF, Template-SVR, and Deep CNN [7] have been compared. Deep CNN is currently one of the best approaches with highest accuracy in eye location. ASEF use the maximum pixel position of convolution result directly to predict eye's location. The Template-SVR approach is formed by replacing the BP network in the proposed model with the nu-SVR implemented by libSVM [24] to draw a fair comparison on the efficiencies of the 2 nonlinear mapping models. ASEF and Template-SVR were implemented in Matlab, while the results of Deep CNN were obtained from [7] with the same training and test data in this paper.

Besides, some leading structure based approaches like CBDS [10], BORMAN [12], and one included in a commercial software LUXAND [25] localizing were also compared. However, their prediction results were obtained from [7]. The test data sets of them were the same to that used in this paper, but the training data are unknown.

#### 5.2.1. Prediction Accuracy

The prediction accuracies of the compared approaches were listed in Table 2. The top 2 results were marked in bold. It should be noted that the robust algorithm usually has high accuracy on LFPW, since samples in this test set were collected in varying situations.

As shown in Table 2, the best results were produced by Deep CNN. However, the proposed method could also achieve similar high accurate results close to Deep CNN. Overall, the proposed method could be the second best one. Averagely, the accuracy of the proposed method was about only 2% lower than that by Deep CNN, while the mean error was 1.1% higher. However, it should be noted that, to achieve such results, the Deep CNN approach should explore the information from 5 feature points with a more complex structure which had 3 cascade levels and several deep models with 3 convolution layers in each level. In comparison, the proposed method only used 2 feature points by a 2-level cascade structure with single convolution layer model in each level. By introducing more feature points for reference with corresponding model structure, there is still large improvement space.

For other texture based approaches, it could be found, without exploration of the global information, the ASEF can hardly work in the complex environment. Considering the supervised learning model, SVR could also well map the relationship between the response map and the eye locations. Nevertheless, the prediction accuracy by nu-SVR is about 4–6% lower than the proposed method by the 4-layer BP network.

In comparison with the structure based approaches, the accuracy of the proposed method is slightly lower than that of CBDS and LUXAND on the BioID by about 0.7%. That might be because of the fact that the samples in BioID are all the regular front face image, which is suited for such structure based approaches. While on the LFPW where pose changes and occlusion commonly exist, the proposed method was better than these methods. Moreover, the mean error of the proposed method was obviously lower than the structure based approach, which also demonstrates the robustness of the proposed method.

#### 5.2.2. Prediction Rate

As mentioned before, the structure based approach employs an iterative prediction approach. The iterative time is not stable and highly related to the study case and initialization. For the situation with side face or occlusion, it may cost seconds to process one image.

Therefore, the experiment on prediction rate was focused on the texture based approach. To simplify the analysis, the face detection time, which is highly related to different situations, was not considered in this experiment. The rate is only calculated for the prediction from the detected face image with the same size. The prediction rate of the Matlab implemented ASEF, nu-SVR, and the proposed method were recorded. Result of the C++ coded Deep CNN was the referenced data.

As shown in Table 3, without nonlinear BP network, ASEF could achieve the highest prediction rate by about 66 fps. The prediction rate by nu-SVR is the lowest with 0.82 fps. In comparison, by the BP network, the proposed approach could achieve 10.5 fps. The well optimized C++ Deep CNN had reported a prediction rate of 8.3 fps which is close to our method.

#### 5.2.3. Training Time

For the structure based approach, usually a well structure model can be obtained by limited samples without too much training time. However, most of the structure based approaches are not as robust as the high accuracy texture based approach. Texture based approach with simple model could be trained rapidly. In the experiment, training of the ASEF with more than 13,000 samples only costs about 200 seconds. However, it is hard to obtain notable improvement through extensive training for those simple models. Therefore, the comparison was focused on the high accuracy texture based approach.

To obtain the above-mentioned accuracy, the proposed method with 13,466 training samples was only trained in 15 minutes including the training of 3 eye templates and 3 BP network in the 2-level cascade model. The low training cost makes our model easy to be tuned and suitable for online learning task.

In comparison, training of other high accuracy models was very slow. The training cost of CNN with back-propagation will be dramatically increased with additional layer. With 3 cascade levels and several deep models with 3 convolution layers in each level, there are totally more than 160,000 parameters to be trained for eye localization. Training of the Deep CNN could cost hours and even days. Due to the long training time, selection of an optimal configuration (like the number of layers and kernels, kernel size, etc.) for the deep model will become difficult. Searching of an optimal configuration will also increase the cost of the training. The nu-SVR model is also time-consuming. For the training of the Template-SVR approach, this costs several hours. Optimization of those models is therefore difficult.

## 6. Conclusion

The stable response distribution by convolution of the face image and eye templates has been discovered. This distribution pattern is beneficial to the eye location. A novel eye location approach has been proposed by learning the distribution of the convolution response maps. The proposed approach only used one convolution layer with a specific eye template and a BP network. In comparison with many state-of-the-art approaches, comparable best prediction accuracy could be achieved by the proposed method with high prediction rate and less training time. The proposed method, which is robust to the pose changes, distortion, and occlusion, can be well used in the complex environment. It has been demonstrated that with proper selection of the template as the convolution kernel, a shallow convolution model could produce similar accurate results to that by deep convolution models with high prediction rate and greatly reduced training time.

In the future work, localization of other critical facial points with the proposed approach will be studied. Efficiency of the kernel selection in the convolution networks will be further analyzed.

## Figures and Tables

**Table 1:** Accuracies of the involved schemes.

Method	BioID		LFPW	
	Right eye	Left eye	Right eye	Left eye
M1	95.7%	96.6%	95.5%	95.4%
M2	96.8%	96.1%	94.7%	94.8%
M3	96.7%	96.8%	96.1%	95.8%
M4	94.2%	94.8%	93.2%	92.7%
Standard	**98.1%**	**98.2%**	**96.8%**	**96.9%**

**Table 2:** Comparison of accuracies among the state-of-the-art eye localization approaches. Note the robust algorithms with high accuracies on LFPW.

Method	Accuracy				Mean error			
	BioID		LFPW		BioID		LFPW	
	Right eye	Left eye	Right eye	Left eye	Right eye	Left eye	Right eye	Left eye
Our method	98.1%	98.2%	**96.8%**	**96.9%**	**2.7%**	**2.4%**	**3.4%**	**3.1%**
Deep CNN	**99.9%**	**100%**	**99.1%**	**99.4%**	**1.7%**	**1.5%**	**2.1%**	**2.0%**
ASEF	1.2%	0.2%	2.4%	0.6%	121.4%	88%	81.2%	99.2%
nu-SVR	96.1%	95.9%	92.8%	92.8%	4.2%	4.1%	4.9%	4.9%
BORMAN	79.1%	75.8%	78.2%	92.8%	7.1%	7.8%	7.8%	8.8%
CBDS	97.7%	**98.9%**	87.9%	91.9%	4.1%	3.9%	7.2%	7%
LUXAND	**98.9%**	98.66%	95.6%	96.8%	4.1%	3.7%	5.6%	4.5%

**Table 3:** Prediction rates of the texture based approaches.

Method	ASEF	nu-SVR	Deep CNN	Our method
fps	66.7	0.82	8.3	10.5
